# Implementation and evaluation of an individualized physical exercise promotion program in people with manifested risk factors for multimorbidity (*MultiPill-Exercise*): a study protocol for a pragmatic randomized controlled trial

**DOI:** 10.1186/s12889-022-13400-9

**Published:** 2022-06-13

**Authors:** Simone Schweda, Gerhard Müller, Barbara Munz, Gorden Sudeck, Peter Martus, Katja Dierkes, Inga Krauss

**Affiliations:** 1 Department of Sports Medicine, Faculty of Medicine, University Hospital, Medical Clinic, Hoppe-Seyler Str. 6, 72076 Tuebingen, Germany; 2 Interfaculty Research Institute for Sports and Physical Activity, Tuebingen, Germany; 3 Allgemeine Ortskrankenkasse AOK Baden-Wuerttemberg, Presselstrasse 19, 70191 Stuttgart, Germany; 4 Department of Sports Science Tuebingen, Faculty of Economic and Social Science, Eberhard Karls University Tuebingen, Wilhelmstrasse 124, 72074 Tuebingen, Germany; 5 Department for Clinical Epidemiology and Applied Biostatistics, Faculty of Medicine, University Hospital, Medical Clinic, Silcherstrasse 5, 72076 Tuebingen, Germany

**Keywords:** Multimorbidity, Physical exercise, Diabetes mellitus Type 2, Hypertension, Osteoarthritis, Overweight, Obesity, Health services research, Cost-analysis, Behavior change techniques, Randomized controlled trial

## Abstract

**Background:**

Multimorbidity is a major problem in Europe, increasing the need for prevention and rehabilitation programs. In Germany no guidelines have been developed that focus on patients with multiple chronic non-communicable diseases (NCDs). Benefits of physical activity (PA) and exercise in NCDs have been proven, but most interventions focus on single conditions. The evaluation of the effectiveness, efficiency and safety of PA programs in patients suffering from multiple NCDs and the feasibility of the implementation within the health care service remain open research questions.

**Methods:**

The multi-site randomized controlled pragmatic trial includes 320 sedentary subjects with at least two of the following NCDs, either manifested or in a pre-stage with evident risk factors: Cardio-vascular disease, Diabetes mellitus type 2, knee/ hip osteoarthritis and obesity. Participants will be recruited from general practitioners and medical specialists and randomized to standard care of a statutory health insurance or *MultiPill-Exercise*. Standard care includes a choice of one or a maximum of two 8- to 12-week health programs, including nutrition, exercise, relaxation or special disease management programs. *MultiPill-Exercise* is based on the bio-psycho-social health model, considering a person-oriented perspective in light of given individual characteristics and context factors. The 24-weeks intervention focuses on aerobic and strengthening exercises in line with the WHO PA recommendations. Psychological and pedagogical elements along with behavior change techniques are implemented to ease the initiation and maintenance of exercise participation and lifestyle change, including nutrition. Primary outcome will be short- and long-term PA measured with the European Health Interview Survey-Physical Activity Questionnaire (EHIS-PAQ). Secondly, the effectiveness of the program on generic, disease specific, economic, and exercise behavioral parameters, as well as program adherence and safety will be evaluated.

**Discussion:**

Results of this trial evaluate the PA intervention program in people with multiple NCDs in a real-life scenario. It will serve as a proof of concept with the opportunity of translation into routine practice. This approach, as a multi-site RCT with its rigorous methods and standardized operating procedures for the conduction of the intervention, will allow valid conclusions for the implementation of PA interventions in people with multimorbidity.

**Trial registration:**

The trial was registered at www.drks.de (ID: DRKS00025033) on 30th September 2021.

**Supplementary Information:**

The online version contains supplementary material available at 10.1186/s12889-022-13400-9.

## Background

Multimorbidity, defined as the co-existence of two or more chronic diseases within an individual [1], is one of the major problems for the health care systems of industrial nations. A German study has shown that in the age group of 40–85 years, 46% suffer from two or more simultaneous chronic conditions, and the incidence of multimorbidity increases further as age increases [2]. Thus, multimorbidity is not the exception, but rather the rule in primary care. The most common chronic conditions are cardio vascular diseases (CVD), diabetes mellitus type 2 (DMT2), obesity (OB) as well as musculoskeletal disorders [3, 4]. Suffering from multiple chronic conditions is most often accompanied by medical complications and a reduction in the quality of life. Mental, physical and social restrictions are common challenges that have to be faced [2, 3]. Multimorbidity therefore puts high demands on the health system. In 2016, € 365.5 billion have been spent on health costs in Germany. Thereof, 14% were caused by CVD, 10% accrued by musculoskeletal disorders and a further 4.6% incurred by endocrine, nutrition and metabolic illness. In the course of the age demographic change the expenses are expected to continue to increase [5].

Multimorbidity has not received much attention from policy-makers so far [3], but the rapid increase of costs and number of people with chronic conditions has intensified the effort on the European and National level to conceptualize and evaluate models that challenge the ever-increasing demands of multimorbidity. Up to now, most of the interventions have focused on single diseases: several disease management programs (DMPs) have been generated, but they do not meet the demands of multiple chronic conditions. Research priorities should therefore be placed on the development and evaluation of effective and cost-efficient treatment strategies geared towards counteracting the aforementioned increasing burden of multimorbidity on the individual and the health care system [2, 6].

The above-mentioned diseases have one common risk factor: chronic inflammation [7–9]. At early stages, conservative therapeutic interventions are important to reduce the inflammatory process and thus prevent progression [9, 10]. To date, the most common conservative treatments for patients suffering from multimorbidity have been pharmacological therapies, although the use of medication is known to have significant side-effects [2]. A non-pharmacological treatment option, physical activity (PA), is also well known, but so far not prescribed often in everyday practice [11]. In recent years, the effectiveness of PA in the treatment of multiple chronic conditions has been evaluated. Regular PA has been proven to reduce 20–33% of the risk of the occurrence of CVD [12] and builds one of the main pillars of the non-pharmacological therapy of DMT2 [13]. Significant improvements in pain and function – and therefore an improvement in the quality of life – in hip and knee osteoarthritis (OA) by PA have also been evaluated [14, 15]. PA shows treatment effects similar to analgesics and anti-inflammatory drugs, with little side effects [16]. Despite given evidence and consistent recommendations, many people do not achieve the PA recommendations of the World Health Organization (WHO). In Germany, for example, less than half of the adult population achieves the minimum recommendation for health-enhancing moderate-to-vigorous PA. When muscle strengthening exercises are included, only 23% fulfill the recommendations [17] and people suffering from multimorbidity are even less physically active than the general adult population [18].

Beside the health-related relevance of PA, nutrition is another important cornerstone for the treatment of NCDs with an inflammatory component. Malnutrition and obesity induce low-grade inflammation on a systemic level and are therefore related to several metabolic diseases [19]. In addition, obesity is a relevant risk factor for the incidence and progression of knee OA, both from a systemic and biomechanical perspective, due to the increased load on the knee joints [20]. A comprehensive lifestyle intervention with the aim to reduce chronic low-grade inflammation should therefore additionally include a healthy diet [10].

In order to engage inactive people in long-term exercise, systematic physical training is necessary, taking into account individual PA promotion and behavior change strategies [21]. In this regard, personal and structural context factors need to be considered [21, 22]. In terms of personal factors, motor skills and health-related knowledge aimed at building up competencies for a health-effective lifestyle regarding PA should be integrated into the intervention [21]. Furthermore, individual exercise-related motives and goals should be included as personal factors in an exercise promotion program to enable long-term development [23, 24]. While health is often the initial motive for physical activity, it is often not sufficient for long-term exercise participation. This requires a variety of motives and goals, such as experiencing aesthetic movement, enjoying nature, distraction, and social contact [23, 24]. At the structural level, the interfaces between patient and physician and between patient and recreational exercise, as well as social support, access to training facilities and suitable exercise offers close to home are important factors that need to be considered [25]. In order to enable a comprehensive, health-promoting lifestyle, the factors mentioned must also be applied to nutrition.

### Objectives

Evidence is given for the effectiveness of PA in the prevention and treatment of single chronic NCDs, though the literature reveals knowledge gaps regarding the evidence of the effectiveness of PA in people with multimorbidity [26]. The primary aim of this pragmatic trial is to evaluate whether a comprehensive exercise and lifestyle intervention (*MultiPill-Exercise*) can help people to engage in regular PA at the end of a six-month intervention and after another 12 months in a follow-up. We hypothesize that engagement in regular PA – quantified with the EHIS-PAQ – is superior in the intervention group *MultiPill-Exercise* in comparison to usual health care offers. Secondary aims are related to the effectiveness of the program on generic and disease specific health outcomes and disease prevention, health economic outcomes, individual behavioral determinants as well as program adherence and safety. The feasibility of the implementation of *MultiPill-Exercise* into a regular health care setting is monitored by a continuous quantitative and qualitative process evaluation.

## Methods

### Study design

The study will be conducted in the context of health service research. The feasibility of the intervention and an exploratory analysis of its effects on health outcomes were tested in a non-controlled pilot study (DRKS00016702). Results have been used to optimize the current study design, for example through putting more emphasis on nutrition, extending supervision time during the study period (24 weeks of supervised machine-based training instead of only 12 weeks) and integrating videos into the exercise sessions at home. The current study is now designed as a multi-site randomized controlled superiority pragmatic trial with two parallel groups. It evaluates the effectiveness, efficiency and safety of the 24-week comprehensive lifestyle intervention *MultiPill-Exercise* in comparison to the regular health care offer of a statutory health insurance company. Participants will be randomized to intervention and control group with an allocation ratio of 1:1. Measurements will be taken at baseline (t0), three months (t3), six months (t6), 12 months (t12) and 18 months (t18). The study was prospectively registered in the German Clinical Trial Register (DRKS00025033) and is documented according to the completed Standard Protocol Items: The recommendations for interventional trials (SPIRIT) checklist [27] and the Pragmatic-Explanatory Continuum Indicator Summary (PRECIS)-2 checklist [28] are available as supplements (Additional files 1 and 2).

### Study setting

The study will be conducted in the federal state of Baden-Wuerttemberg (BW), Germany. Across the state, four organizational units of a statutory health insurance company, each with up to two associated study sites, were selected. The selected sites are sufficiently distant from each other, covering several areas of the state. Both urban and rural areas are considered in the selection.

### Eligibility criteria

The study population includes males and females of the adult population (> 18 years) who (1) have an active membership in the involved health insurance company, (2) show elevated risk factors or manifested diseases for at least two of the four selected diagnoses, (3) show constant medication during the previous 3 months and (4) are currently insufficiently active. Detailed inclusion and exclusion criteria are listed in the table (Table 1) below.

**Table 1:** Inclusion and exclusion criteria

**Inclusion criteria**		**References**
**Administrative**	- Insurance holder of the involved insurance company in the past two or more years prior t0^a^	
**2–3 of the following diagnosis and/or risk factors:**		
**Diagnosis**	**Risk Factor**	
Osteoarthritis of hip and/or knee:	- Self-report of physician diagnosed life-time prevalence of knee and/or hip osteoarthritis	[29]
Diabetes Mellitus Type 2:	- German diabetes risk score (GDRS) ≥ 57 points	[30, 31]
Cardiovascular:	- PROCAM-Score: 10-year myocardial infarct risk > 10% in the age- and sex-related reference group	[32]
Overweight/Obesity:	- BMI ≥ 27 kg/m^2^	[33]
**Ongoing Therapy**	- No or constant medication^b^ during the previous three months	
**Exclusion criteria:** (one or more positive answers lead to non-eligibility)		
Overall	- End organ damage^c^ - Physical activity before the intervention meets more than 75% of the national physical activity recommendations for adults - Self-reported personal fitness level is under 20 min of brisk walking - Injuries/diagnoses that do not allow the use of the strength machines (acute herniated intervertebral disc, acute fractures, recent spinal surgeries, glaucoma with elevated intraocular pressure)	
Osteoarthritis patients only:	- Appointment for elective joint replacement	
Cardiovascular patients only:	- Patients diagnosed with heart failure are excluded	
Overweight/Obesity patients only:	- Physical prerequisites for training on the equipment are not given	

^a^ in case of recruitment difficulties due to this restriction, it will be reduced to a previous insurance period of 18 months

^b^ this refers to the medication in relation to the diagnoses of interest

^c^ damage of an organ caused by the primary condition requiring medical or invasive treatment of a physician (i.e. Stent, myocardial infarct, diabetic foot, gastric band, TEP)

*PROCAM-Score* Prospective cardiovascular Muenster Study, t0: Baseline assessment

### Recruitment

**Fig. 1:**
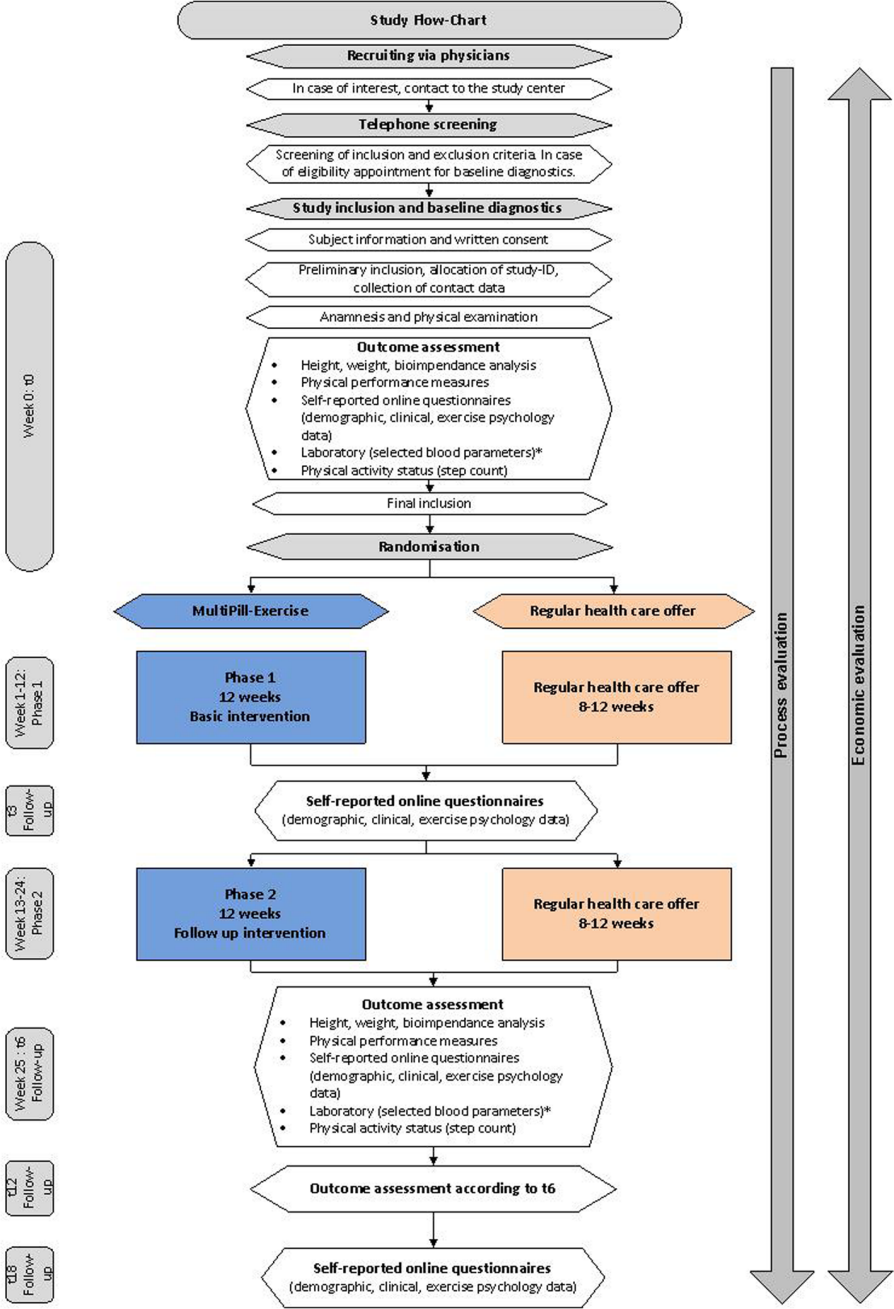
Study Flow-Chart; *corresponding parameters are only collected for a subgroup *n* = 60; t0, t3, t6, t12, t18: months after baseline diagnostic

The recruitment period will be 8 months and will be conducted in two waves due to organizational reasons related to the conduction of the intervention. In total, the *MultiPill-Exercise* intervention (IG) will be offered to 160 participants, while another 160 subjects will receive standard care (CG). Participants will be recruited via general practitioners and specialists (i.e. internal medicine, orthopedists, diabetologist, cardiologists) during regular consultations. The physicians will be informed beforehand about the study and the new health care offer *MultiPill-Exercise* by the staff of the health insurance company (Physician-Partner-System). In addition, exercise and nutrition specialists from the health insurance company will assist with recruitment by recommending these customers to get in contact with their physician in order to get a prescription, if applicable. Additionally, the commonly used media of the statutory health insurance company (advertisements in own or local magazines) will be used as well. Physicians prescribe the health care offer to potentially eligible patients (green prescription: this is a written recommendation of the physician for remedies that do not fall with budget, e.g., a therapy supplement or exercise advice) and also provide their patients with the study flyer, which includes contact details and other study related information, including a study homepage. If interested, participants contact the study examiners at the university hospital. Initial contact via telephone serves to inform potential participants on study contents, aims and the time-line of the study. Furthermore, inclusion and exclusion criteria will be checked. In case of potential eligibility, an appointment at the participant’s nearest study site will be made, where further information and written consent for the study will be obtained. For a sub-sample only, laboratory data (blood, urine) will be assessed. If interested and eligible, further consent will be acquired. At the study site, final checks for the inclusion and exclusion criteria will be undertaken by the study team. Participants who do not meet the inclusion criteria after the personal appointment are offered reference to the health insurance company's exercise specialists, who will provide them a different health service offer on a voluntary base. In the case of preliminary study inclusion, study-IDs are assigned. The IDs contain a number for the study center in addition to a sequential number. The subject then undergoes baseline medical examinations and physical performance tests. If no exclusion criteria are found during these examinations, the subject is considered to be fully included into the study and will be randomized into the study interventions as outlined below. Participants randomized into IG will be allocated to the *MultiPill-Exercise* intervention. They will immediately receive appointments and necessary information about the study from the study team. The participants of the CG will be assigned to the staff of the health insurance company, who will explain the possibilities of standard care at the particular study site to them. The standard care intervention will then be determined, taking into account the customer’s preferences. Details are depicted in the study flow-chart (Fig. 1).

### Randomization

**Fig. 2:**
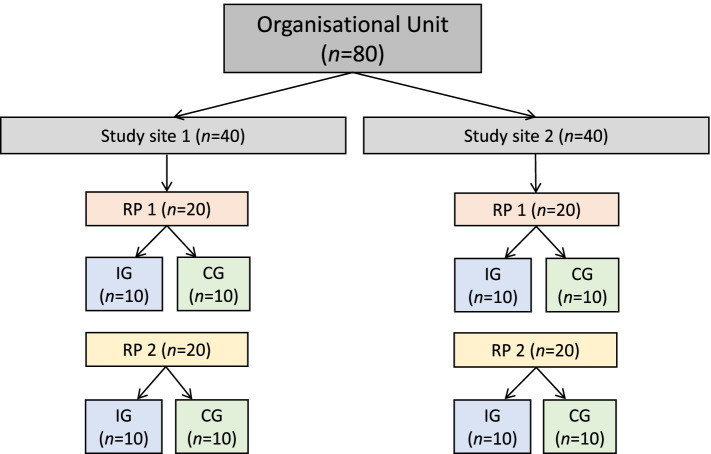
Randomization process; Level 1: Organizational unit (head of the associated study sites); Level 2: Study site (allocation to study site is based on proximity); Level 3: Recruiting phase (RP) in red is the first (RP1) and yellow the second (RP2) phase; Level 4: Randomization group (intervention group (IG) in blue and control group (CG) in green

The study staff retrieve the study arm of a new subject online from the study software SecuTrial, a web-based database application for administrative, organizational and implementational support in clinical and epidemiological research projects, hosted by the statistical center of the study. For practical recruitment reasons, two identical intervention/control phases will be conducted at each of the study sites. For each phase and site, approximately *n* = 10 subjects will be allocated to IG and CG, respectively. As such, randomization will be generated in two sequences (= phase), each with eight strata (= study site) with random block lengths of four or six and a balanced allocation of *n* = 20. For each study site, a different randomization list will be generated, and a new randomization list will be used for the second study phase. Figure 2 displays the randomization process, explanatory for one organizational unit.

### Blinding

Due to the design of the study, blinding of the subjects is not possible as treatment exposure is evident. Assessors will not be blinded. For the questionnaires, blinding is not applicable, as these outcome measures are self-administered by the participants. Exercise specialists and providers will not be blinded to the allocation groups, as the realization of the ‘new’ intervention is evident. But data analysts will be blinded from t0 to t6 but not at later time points. The data analysts receive all data under the study-ID. The re-identification of the study-ID with the name and contact information of the participants is only possible for the study leaders at the university hospital and an independent party involved in the data privacy concept.

### Participant timeline and collection points

**Fig. 3:**
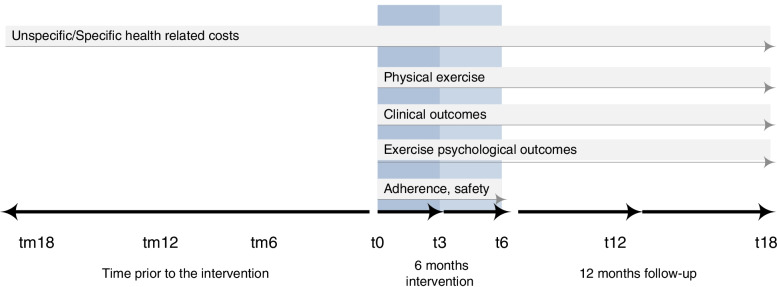
Collection points and outcome measures; t = collection point, the number relates to the months of collection point related to baseline (t0); m = minus; the darker blue box represents intervention phase 1, the lighter blue box intervention phase 2 for the IG

First patient inclusion is planned for April 2022. Assessments will be conducted at the participant’s nearest study site, with exception of two local sites where the examination will be performed at the university hospital, at baseline (t0), after the intervention (t6) and after another six months follow-up period (t12). Additional to the on-site assessments, online questionnaires will be sent at t3 and t18. Economic evaluation will further compare data at 6 (tm6), 12 (tm12), 18 (tm18) and 24 (tm24) months, retrospectively (m = minus) prior to t0 (Fig. 3). All participants are expected to have completed the intervention period in early 2023. All participants are expected to have completed the study by the end of March 2024.

## Interventions

Both types of interventions (regular health care offers for CG, *MultiPill-Exercise* for IG), are conducted at the health centers of the statutory health insurance company, in which different kinds of health care programs are offered to customers of the insurance company on a regular basis.

### Person-oriented comprehensive exercise program “*MultiPill-Exercise*”

**Fig. 4:**
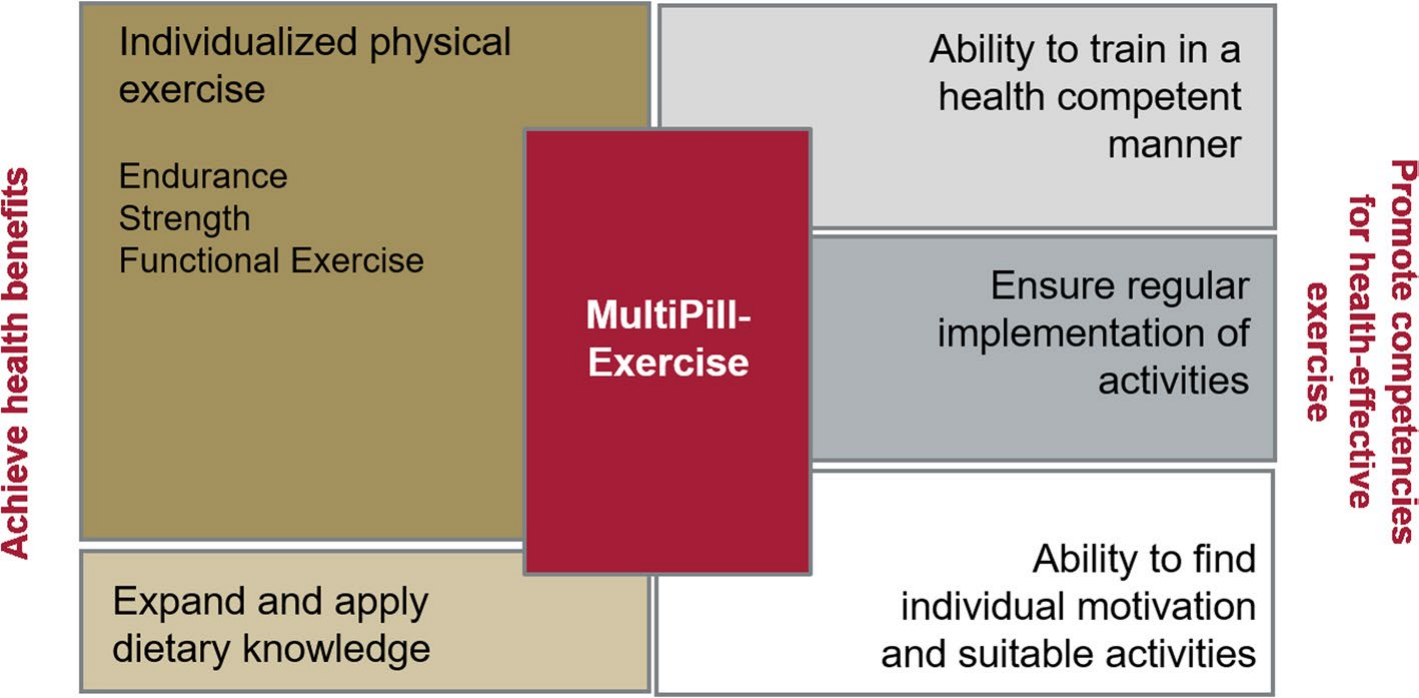
Intervention modules of *MultiPill-Exercise*

The concept of *MultiPill-Exercise* is based on the biopsychosocial health model of the International Classification of Functioning, Disability and Health (ICF) [34]. Figure 4 displays the different modules of the intervention.

The biological perspective relates primarily to a lifestyle intervention comprising exercise and nutrition to enhance health benefits that can then mediate participation and social well-being. The individualized exercise intervention is designed according to national and international PA recommendations (150 min of moderate intensity endurance training or 75 min vigorous endurance training or a combination of both and additional muscle strengthening exercises twice a week [35, 36]. Healthy nutrition relates to the quantity and quality of energy consumption, with the aim of regulating weight and counteracting systemic inflammation. The psychosocial aspects of the intervention also consider the personal and structural context factors of the participants. Motives for exercise participation are specifically considered to ease the initiation and maintenance of regular exercise [23]. In addition, further person-related supporting factors as well as barriers are included to individualize the program. It’s not only about doing health enhancing activities, but also about gaining domain-specific health competencies to allow self-determined lifestyle change over the long-term. Health competencies are fostered by educational and psychological strategies, such as the connection of “learning”, “acting” and “reflecting”, or familiarization with behavior change techniques to ease self-regulation. More details on the conceptualization of the intervention are described elsewhere [37].

The intervention is divided into two phases, each lasting 12 weeks, and each with a decreasing amount of supervision and guidance. Throughout the entire study period, a maximum of three on-site appointments/week with a maximum duration of 90 min each will be scheduled. The intervention includes three overarching types of components: (1a/b) Exercise, divided into two phases of 12 weeks; (2) patient education sessions (including dietetics also), focusing on knowledge transfer; and (3) systematic usage of behavioral change techniques. An overview of the detailed intervention design can be found as a supplement (Additional files 3 and 4).

- The exercise training of the first 12 weeks will include four progressive phases including different training methods and principles (Additional file 3 shows the associated training specifications). Individual and group trainings, indoor and outdoor, on-site and home-based sessions will take place. The duration of the exercise sessions will vary between 45–60 min.

**Table 2:** Outcome measures

Characteristics & confounders	Description and instrument	Data source	Sample	Collection points
**Overall**				
Participant’s characteristics	Date of birth, gender, ethnicity, BMI (height, weight), highest academic qualification (no degree, middle school, high school), labor situation (working, retired, unemployed in rehabilitation status), current profession, weight at birth, week of pregnancy at birth	SAQ	IG, CG	t0
Clinical status	Diagnoses of OA, Diabetes mellitus Type 2, Cardio vascular disease, and if applicable, date of initial diagnosis, in case of OA site (s) and joint (s) of OA diagnosis	SAQ, on-site CRF	IG, CG	t0
	Other diagnosed comorbidities	SAQ, on-site CRF	IG, CG	t0
	Current medication assessed via medication schedule and/or packaging. Also, pre-determined drugs are recorded via the IDB	SAQ IDB On-site CRF	IG, CG	t0, t6, t12
History of exercise participation	Athleticism during childhood and adolescence	SAQ	IG, CG	t0
Outcome expectations	Personal therapy expectations	SAQ	IG, CG	t0
**Primary Outcome measure**	**Clinical Outcomes**			
Physical activity status	Leisure-time physical activity in a typical week (EHIS-PAQ, HEPA Index [38])	SAQ	IG, CG	t0, t3, t6, t12, t18
**Secondary outcome measure**	**Clinical outcomes**			
Physical activity status	Work-related and transport-related physical activity in a typical week (EHIS-PAQ [38])	SAQ	IG; CG	t0, t3, t6, t12, t18
	Actigraph (wGT3X-BT Accelerometer) for 7 days	wGT3X-BT Accelerometer	IG, CG	t0, t6, t12
Health related quality of life	Veterans RAND 12 Item Health Survey (VR12), retrospective 4 weeks [39, 40]	SAQ	IG, CG	t0, t3, t6, t12, t18
Global health assessment	One Item, General Health, of the VR12 [39, 40] to assess global health [41]	SAQ	IG, CG	t0, t3, t6, t12, t18
Overall perceived benefit	Overall perceived satisfaction with the benefit of the intervention [42]	SAQ	IG, CG	t3, t6, t12, t18
Osteoarthritis specific outcomes	Hip Osteoarthritis Outcome Score (HOOS), Knee injury and Osteoarthritis Outcome Score (KOOS)	SAQ	IG, CG (only participants with OA)	t0, t3, t6, t12, t18
	Pain visual analog pain scale (0–10) [42, 43]	SAQ	IG, CG (only participants with OA)	t0, t3, t6, t12, t18
Cardio vascular risk status	10-year risk of myocardial infarction, PROCAM-Score short form [32]	SAQ	IG, CG	t0, t3, t6, t12, t18
Diabetes mellitus risk status	German diabetes risk score [30, 31]	SAQ	IG, CG	t0, t3, t6, t12, t18
Dietary intake	Food Frequency Questionnaire [44]	SAQ	IG, CG	t0, t3, t6, t12, t18
	**Physiological Outcomes**			
Physical performance measures	Spiroergometry: VO_2_ max, respiratory ratio, physical working capacity (Aeroscan GmbH)	Aeroman	IG, CG	t0, t6, t12
	Lower body strength measure: 30 s sit to stand Test (CST) [42, 45]	30 CST	IG; CG	t0, t6, t12
	Electrocardiogram (ECG) (custo med GmbH) to assess heart rate, systolic and diastolic blood pressure at rest and during exercise test	Custo Cardio 300	IG; CG	t0, t6, t12
Body composition	Assessment of body composition (muscle, fat, water distribution) with bioimpedance analysis (SMT medical GmbH & Co.)	BIA101 BIVA	IG, CG	t0, t6, t12
Metabolomics	Capillary blood sample from earlobes on Guthrie cards to assess carnitine, short- and long-chain acylcarnitines, methionine, phenylalanine, tyrosine, valine, leucine, isoleucine, succinylacetone, citrulline	Blood sample	IG, CG	t0, t6, t12
Laboratory data	Fasting blood sample: HbA1c, insulin, fasting glucose, IL-6, CRP, cholesterol, LDL, HDL, triglycerides, miRNAs, adiponectin, leptin, resistin, COMP Urine sample: CTXII	Blood and Urine sample	Sub-sample IG, CG	t0, t6, t12
	**Exercise psychological outcomes**			
Exercise-specific self-efficacy	Multidimensional Self-Efficacy for Exercise Scale: task, coping, and scheduling efficacy [46]	SAQ	IG, CG	t0, t3, t6, t12, t18
Sport and exercise-related self-concordance	Self-concordance of sport- and exercise-related goals: intrinsic, identified, introjected, and extrinsic modes of motivation [47]	SAQ	IG, CG	t0, t3, t6, t12, t18
Physical-activity related health competence	Physical-activity related health competence questionnaire: control of physical load, affect regulation, and self-control [21, 48]	SAQ	IG, CG	t0, t3, t6, t12, t18
Sport- and exercise specific motivational competence	Questionnaire on motivational competence in exercise and sport: knowing one’s own preferences, knowing what to expect in different exercise and sport activities and choosing and arranging an exercise and sport activity [49]	SAQ	IG, CG	t0, t3, t6, t12, t18
Action and coping planning	Exercise planning scales: action plans, coping plans [50]	SAQ	IG, CG	t0, t3, t6, t12, t18
Attitudes towards physical activity	Cognitive and affective attitudes towards physical activity [51]	SAQ	IG, CG	t0, t3, t6, t12, t18
Motives and goals for exercise	Bernese motive and goal inventory [52]	SAQ	IG	Throughout intervention phase 1 (week 7), t6, t12
Fear of movement /(re)injury	Tampa Scale for Kinesiophobia [53]	SAQ	IG, CG	t0, t3, t6, t12, t18
	**Economic Data**			
Unspecific and specific (disease-related) health care costs	Outpatient costs, hospital costs, medication, adjuvants, rehabilitation treatment, medically prescribed supplementary treatments (e.g. hydrotherapy, physiotherapy, massages)	IDB	IG, CG	tm18, tm12, tm6 t6, t12, t18
Unspecific/specific (disease related) periods of disability	Days of disability (overall and related to referred diseases)	IDB	IG, CG	tm18, tm12, tm6 t6, t12, t18
Intervention related costs	Costs for human and physical resources/session	IDB	IG	t6, t18
	**Process evaluation**			
Characteristics of the usual care arm	Questions on utilization of usual care offers	SAQ	CG	t3, t6, t12
Participants’ satisfaction	Modified version of the ZUF-8 Questionnaire to assess participants’ satisfaction with the intervention [54]	SAQ	IG, CG	t3, t6, t12, t18
Participants’ view on the intervention	Qualitative interviews with participants including questions on the appropriateness and quality of the intervention delivery	QI	Sub-sample IG	after last intervention delivery
Treatment fidelity	Field notes for intervention progress (Troubleshooting)	Documentation sheets	Research Team	continuously
	Notes for intervention delivery	Notation sheets	Interventionists	continuously
Characteristics of interventionists	Questionnaires on occupational self-efficacy [55], subjective value attributions [56] and implementation outcomes (acceptability, appropriateness, and feasibility of the intervention) [57]	SAQ	Interventionists	t0, t6
Interventionists’ view on the program	Qualitative interviews with exercise specialists regarding acceptability, practicality, appropriateness and fidelity aspects of the intervention delivery	QI	Interventionists	after last intervention delivery
	Satisfaction with the interventionists’ training [58]	SAQ	Interventionists	after interventionists training
Evaluation of implementation regarding interfaces of the health service innovation	Practitioners’ view on the acceptability, appropriateness, and feasibility of the program and its prescription [57]	SAQ	Health insurance company (APS)	during recruitment
	Mode of access to the program	Telephone screening	IG, CG	during recruitment
Evaluation of implementation regarding the interface with community-based exercise offers	Qualitative interviews with participants and employees of the health insurance company	QI	Sub-sample IG and interventionists	after last intervention delivery
Adherence to exercise training	Summarized number of attended training sessions and reported trainings sessions according to training logs of the exercise specialists and participants (IG)	Training logs, on-site reporting sheet (IG); SAQ, IDB (for CG only)	IG, CG	t3, t6
	**Safety and Adverse Events**			
Adverse events and side-effects	Summarized number and details of adverse events and side-effects according to training log and case report form	Training logs, on-site CRF, SAQ	IG	t3, t6
**Covariates**				
Depressive and anxiety symptoms	Self-report of depressive symptoms and anxiety symptoms in the last week [59]	SAQ	IG, CG	t0, t3, t6, t12, t18
Osteoporosis risk status	German version of osteoporosis risk test [60]	SAQ	IG, CG	t0, t3, t6, t12, t18
Back pain	Three months retrospective questionnaire on the severity of chronic back pain [61, 62]	SAQ	IG, CG	t0, t3, t6, t12, t18

*APS* Physician-Partner-Service, *CG* Control group (regular care), *CRF* Case report form (on-site CRF will be provided by study staff, exercise specialists and physician, if applicable; *eCRF* Electronic case report form), *ECG* Electrocardiogram, *HEPA Index* Health enhancing physical activity, *IDB* Insurance data base, *IG* Intervention Group *MultiPill-Exercise*, *PE* Physical exercise, *SAQ* Self-administered (online) questionnaire, *t* Collections points, *m* Minus. 0, 3, 6, 12, 24: month of collection point related to baseline (t0), *QI* Qualitative interview

The initial two weeks will focus on familiarization with exercise and motor learning. The participants will also be introduced to training at the study sites including strength training on the weight-machines and ergometers and tread-mills for endurance training, where applicable. For functional strength training at home, videos including whole body exercises in different starting positions and difficulty variants will be made available to participants by logging into an online platform from the health insurance company. The following four weeks (week 3–6) will emphasis basic endurance training (cardio-vascular training, machine-based and functional strength training). For the endurance training, different training methods will be introduced, such as the continuous method and high intensity interval training. Machine-based strength training in this phase is performed as strength endurance training. The subsequent continuation training (week 7–10) will carry on the goals of the previous weeks. However, by reducing the number of repetitions and increasing the intensity, the machine-based training will be changed to a targeted muscle-building workout. Functional training sessions will be supplemented by exercise group offers close to everyday life ("movement teaser") that focus on different motives for exercise participation (health, aesthetic, competition, contact, enjoyment, distraction, nature etc.). The final maintenance training of the first phase (week 11–12) is designed to promote independent exercise participation and will include only one supervised training session/week.

1. During the second 12 weeks, the activities of the first phase are to be maintained. Participants will continue machine-based training sessions 1x/week at the study sites. The endurance sessions can be done independently home-based or in a group. Cooperation with local sport clubs and gyms or else will be encouraged.

Each participant will receive a weekly individualized training plan in both phases of the intervention, which will include training parameters in addition to the scheduled training sessions. For endurance training, heart rates and watts are added individually, calculated from the performance diagnostics. Strength training will be regulated by the Borg scale (0: no effort- 10: maximum possible effort) (details see Additional file 3).

(2) In order to strengthen the effect and action knowledge of the participants, participant education sessions of 30 min each will be held, delivering theoretical and practical knowledge on the basics of training sciences, nutrition (healthy and anti-inflammatory diet), themes to enhance motivation and volition [63], motives and goals of PA [23, 52] and PA in daily life [64]. While five theory sessions will be scheduled for phase 1, phase 2 will include a nutritional booster session (anti-inflammatory diet). A theory–practice link is always strived for. 

(3) Three individual counselling sessions (30 min each) using further behavioral change techniques will take place in phase one. The individual counselling to enhance regular sport participation will be conducted by a guide; an exercise specialist who will also act as a personal contact person. The guide will be briefed on the diverse behavior change techniques included (MoVo-Lisa concept [65], the exercise counseling approach based on motives and goals in exercise and sport (COMET) [23], including 5A’s [66], and techniques from Motivational interviewing [67]). The personal guide will also be responsible for weekly feedback on the participant’s training logs. Phase two includes two individual counselling appointments (anti-inflammatory diet and COMET). The counselling sessions are based on the individual training diaries, the participant’s feedback on the movement teasers and the individual results of the assessment of the Bernese motive and goal inventory in exercise and sport (BMZI) [52]. The counselling on nutrition and dietetics will build on two seven-day nutrition logs (these are kept after each of the nutrition lectures) and will be evaluated and discussed with a nutritionist from the insurance company.

All individual and group sessions on-site will be conducted by exercise professionals, employees of the statutory health insurance company at each of the study sites. Several exercise specialists and nutritionists from the statutory health insurance company will be trained in advance on the *MultiPill-Exercise* intervention design. Only trained exercise professionals will also act as guides in the intervention. The exercise specialists will receive a comprehensive manual for delivering the exercise intervention to enable standardization across study sites.

### Standard Care (CG)

The standard care of statutory health insurance company offers every insured person the opportunity to participate in health offers, provided and conducted by employed professionals from the health insurance company, free of charge. For a more detailed information of the German health care see Struckmann, Boerma [68].

Participants of the CG are requested to select from the standard care offers of the statutory health insurance company. Potential offers are in the field of nutrition, exercise and special DMPs, general health and ‘fit and active’. The exercise offers last 8–12 weeks. Participants of the CG will be provided with options by an exercise specialist of the insurance company and will then be able to enroll in the selected offers. Over the study period, participants can attend two different offers, as foreseen in regular care. The combination and choice of offers can be freely arranged according to the participants' own preferences. An exception applies to participants with a physician’s recommendation for prevention programs. With this recommendation, they can also take part in additional offers. All offers will be provided by exercise specialists from the statutory health insurance company and are based on their own manual, which are adhered to in a restrictive manner. Nutrition offers include: a workshop that focuses on education, particular recommended foods and the right combination of foods to induce positive health effects. It also highlights general principles of a balanced diet and practical tips, such that the knowledge can be implemented in the kitchen. The second offer, ‘body and soul in balance’, fosters active weight loss and mindful eating. The aim here is on sustainable change in diet, with more desire for exercise and fun within a lifestyle that relies on personal responsibility, knowledge and conscious decisions. The disease specific DMPs for CVDs include: ‘All the best for your heart’. This offer teaches the basics of avoiding and reducing coronary heart disease. ‘Fourfold increase in quality of life’ (DMP for DMT2) aims to increase everyday activities, giving information on avoiding obesity, recovery from physical and mental stress and information on special foot care for diabetes. Also, light gymnastics, light walking and a special strength-endurance training can be chosen. A focus of the general health offers is to reduce physical inactivity and to strengthen physical health resources. The offer includes courses to increase fitness, flexibility, endurance and strength. Included courses are: Clever-Walking, Fit-Mix, Functional Training, Easy Running, Outdoor Fitness, Back-Fit, and Back-Power.

### Process evaluation

The chosen pragmatic randomized controlled trial design is intended not only to evaluate the effectiveness of an intervention, but also to provide information on how the intervention works and how the intervention and its implementation in its specific context are influenced by various barriers and facilitating factors [69, 70]. Particularly for complex interventions such as *MultiPill-Exercise*, which are composed of multiple interacting components, determining the causes of change (primary action mechanisms) and identifying conditions for success (contextual factors) are key. Consequently, an accompanying process evaluation will be conducted with the aim of understanding not just if, but how and why an intervention has a particular effect, and which parts of a complex intervention have the greatest impact on outcomes [70, 71]. Thus, the process evaluation will help to understand the relationship between specific intervention elements and intervention outcomes, and will provide important information about key aspects that should be given special attention in the (successful) implementation of *MultiPill-Exercise* into regular health care [72]. For this purpose, implementation aspects from the perspective of therapists, participants, study sites and involved organizations will be identified in addition to the primary outcomes and mechanisms of action. Assessment procedures will be described in more detail in the following sections.

### Outcome measures

All participant-reported outcomes will be assessed using SecuTrial online questionnaires (InterActive Systems Berlin) at baseline and all follow-up time points. All physical performance measurements will be assessed on-site at t0, t6, t12 only (Fig. 3 and Table 2). The assessor team will consist of members of the study team, composed of employees of the university and university hospital, as well as a physician of the University hospital at t0. All assessors will be trained in advance and the examinations will follow the same order and predefined standard operating procedures (SOPs). Demographic data, clinical status (details of relevant history of disease, medical history, medications), history of exercise participation and outcome expectations are assessed in addition to primary and secondary outcomes. All on-site data will be directly entered to the electronic case report form (eCRF) using the software SecuTrial. The software automatically performs plausibility queries when the data is saved. The participants will receive E-Mails prior to the data collection, reminding them of the upcoming data collection.

#### Primary outcome measure

The retrospective European Health Interview Survey—Physical Activity Questionnaire (EHIS-PAQ) will be used to assess PA. The 8-item questionnaire covers physical activities during work, transportation, and leisure time, including sports activities, aerobic health enhancing activities and muscle-strengthening activities, over a typical week [73]. As an indicator that estimates compliance with the WHO aerobic PA guidelines, information on transportation-related PA (excluding walking) and leisure-time PA can be combined. The health enhancing physical activity (HEPA) Index [73] will be the primary outcome. EHIS-PAQ has acceptable reliability and moderate to strong validity for all domains other than moderate-to vigorous PA [74]. Clinical relevance is ensured by basing the outcome measure on (inter-) national physical activity recommendations.

#### Secondary and other outcome measures

##### Physical activity status

Further data from the EHIS-PAQ such as overall PA, including transport-related PA and especially the number of muscle-strengthening sessions, will be taken into account in order to analyze the fulfilment of health-oriented recommendations for aerobic PA and muscle strengthening activities (dichotomous outcomes: yes or no).

In addition to the self-report measure of PA, we will collect objective, device-based measurements of the physical activity status. For this, all participants will be asked to wear an accelerometer (wGT3X-BT Accelerometer) on a belt on the waist on the right hip during daytime for seven consecutive days after the on-site examinations [75, 76]. Participants will be included in the final analysis if at least three days of 10 h/day of wearing time is recorded. Instructions on handling of the device will be given by a trained research member. To return the device after the exposition week, a stamped and addressed envelope will be given to the participants.

##### Health related quality of life

To measure health related quality of life, the adapted and validated German version of the Veterans RAND 12-Item Health Survey (VR-12) will be used [77]. The four-week retrospective questionnaire can be summarized into two parent scales (1) Physical Component Scale and (2) Mental Component Scale each derived from four different health domains ((1): physical functioning, role physical, bodily pain, general health), ((2): (vitality, social functioning, role-emotional, mental health). The question on general health is further used as an one-item instrument for patient global assessment [41].

##### Overall perceived benefit

The overall satisfaction with the perceived results of the intervention will be evaluated using a one item question with a 5-point Likert scale according to Rolfson, Wissig [42].

##### Disease specific outcomes

To assess the status of knee and hip OA, the German versions of the Knee Injury and Osteoarthritis Outcome Score (KOOS) [78] and Hip Dysfunction and Osteoarthritis Outcome Score (HOOS) [79] will be applied to the participants with knee and/or hip OA. Both questionnaires consist of 5 subscales: pain, symptoms, activity of daily living, function in sport and recreation and knee or hip related quality of life. The disease-specific instruments are valid, reliable, easy to complete and simple to score [79, 80]. In addition, the participants will be asked to rate their pain during the last four weeks on a visual analog pain scale.

The Prospective Cardiovascular Münster (PROCAM)-Score is a simple and effective way to assess the risk of acute coronary events among adults [81]. Participants with a 10-year myocardial infarct risk > 10% when compared to the reference group are listed as participants at risk for CVD. For the study, the shorter version of the questionnaire will be used which includes: age, weight, height, systolic blood pressure, gender, smoking status, family disposition, current drug treatment of hypertension, DMT2 status [82].

To assess the risk for developing DMT2 within five years, the German Diabetes Risk Score (GDRS) will be used [30, 31]. This noninvasive measurement showed an excellent discrimination in detecting prevalent undiagnosed DMT2. A score of  ≥ 57 points indicates an increased risk of developing diabetes in the next 5 years.

##### Dietary intake

The food frequency questionnaire (FFQ) details the frequency and portion size of common consumed foods. In total, 29 food groups are assessed. The FFQ used in this study was developed for the German Health Examination Survey for adults 2008–2011 (DEGS) [44]. For the German adult population it shows reasonable validity [44].

##### Physiological outcomes

Spiroergometry will be performed to determine the maximum oxygen (O_2max_) uptake, the respiratory ratio and physical working capacity. Therefore, bicycle ergometry will be conducted (incremental test until exhaustion, duration of each increment: 2 min, start at: 0,5 watts x body weight in kg, increment: 0,3 watts x body weight in kg). The breathing gas analysis is carried out using Aeroman professional (Aeroscan GmbH, Berlin). For this purpose, the participant breathes through a breathing unit for 30 s at the end of each stage. A 12-channel resting ECG (custo cardio 300, custo software, custo med GmbH) after 5 min rest will be performed before the performance test to rule out abnormalities and to assess resting blood pressure. Also, during the entire test, an ECG will be recorded and supervised by a physician at t0. Reasons for immediately stopping the ergometry include chest pain, systolic blood pressure > 200 mmHg, ECG abnormalities, intolerable dyspnea and cramps. In addition, capillary blood will be dropped onto a Guthrie-card during performance diagnostics (before and after exercise) for all participants. These serve to record various metabolic markers.

Lower body strength and leg strength endurance will be measured with the 30-Second-Chair-Stand Test. For the test, the participant sits in the middle of a chair, hands clasped in front of the body, feet flat on the floor and back straight. The test person will be asked to stand up, in a completely upright body position, and to sit down on the chair again as many times as possible within 30 s. Depending on gender and age, the minimum number of repetitions will be graded [45].

Body composition will be assessed with bioimpedance analysis (BIA 101 BIVA, SMT medical GmBH & Co). In a lying position, two skin electrodes will be attached to the hands and feet on one side of the body and connected to the BIA101 BIVA via a thin measuring cable. The measured values appear on the device display and will be transferred wirelessly via Bluetooth to the Bodygram PLUS software.

For a predefined sub-sample only (*n* = 20 IG, *n* = 20 CG), laboratory parameters (urine, blood) will be assessed. Subgroup laboratory data analysis include standard laboratory data, adipokines and osteoarthritis markers, metabolomics and qPCR analysis of miRNAs. Trained staff will collect venous blood in the morning after 12 h fasting. Selected miRNAs as well as various markers for osteoarthritic events (COMP, CTXII) and for metabolic function (leptin, adiponectin, resistin) will be recorded in blood or urine samples. Finally, laboratory parameters that are usually positively influenced by physical training (fasting blood glucose, Hba1c, insulin, IL-6, CRP, cholesterol, LDL and HDL) will be analyzed. Approx. 40 ml of blood will be taken at each of the measurements.

*Exercise psychological outcomes* Exercise-specific self-efficacy will be measured with a questionnaire based on the validated Multidimensional Self-Efficacy for Exercise Scale (MSES) [46], which has already been applied in other exercise studies [83]. The MSES comprises nine Likert-like items assessing the three behavioral subdomains task, coping, and scheduling efficacy on 100% confidence scales.

To capture the concept of sport and exercise-related self-concordance, a German-language instrument for measuring the self-concordance of sport- and exercise-related goals (SSK-Scale) will be applied [47]. The validated 12-item version of the scale captures four 3-item subscales measuring the intrinsic, identified, introjected, and extrinsic modes of motivation on a 6-point Likert scale [47].

According to the model of physical activity-related health competence (PAHCO), we will assess the sub-scales control competence for physical load, competence for PA-specific affect regulation, and PA-specific self-control. We will apply the validated 13-item version of the PAHCO questionnaire, which uses a 5-point Likert scale [21].

Sport and exercise specific motivational competence will be operationalized using a one-dimensional scale developed and validated by Schorno, Sudeck [49]. It measures the self-determined ability to choose a suitable exercise and sport activity. The 4-item questionnaire covers the following three facets of motivational competence on a 5-point Likert scale: knowing one’s own preferences, knowing what to expect in different exercise and sport activities, and choosing and arranging an exercise and sport activity [49].

Action and coping planning will be assessed using the validated exercise planning scales developed by Sniehotta, Schwarzer [50]. It includes four items on each of the two subdomains, action plans and coping plans, on a 4-point Likert scale.

To assess attitudes towards physical activity in its cognitive and affective components, a psychometrically tested German version of the validated questionnaire developed by Crites, Fabrigar [84] will be applied. In this short questionnaire, four items each assess the two components using semantic differentials with both positive (e.g., *healthy*) as well as negative (e.g. *useless*) adjectives included on a 7-point bipolar scale [84].

Motives and goals for exercise participation will be evaluated throughout the first intervention phase (week 7) at t6, t12 and t18 for the intervention group only, using the Bernese motive and goal inventory (BMZI) [85]. The updated version of the questionnaire consists of 23 items covering seven motives and goals (Body/Appearance, Contact, Competition/Performance, Aesthetics, Distraction/Catharsis, Fitness and Health) [86].

To assess fear of movement/(re)injury, the German Version of the Tampa Scale for Kinesiophobia (TSK-GV) will be applied [53]. The 11-item version of the TSK-GV with a 4-point Likert scale has been shown to be a reliable and valid measure. Results of the scale may be predictors for the persistence of pain-related disability [53].

##### Economic data (insurance data base; firm software)

Economic data comprise unspecific and specific disease related health care costs. Intervention related costs for the exercise program will be taken into consideration as well.

Unspecific health care costs (overall costs): Outpatient acute care, hospital costs, rehabilitative treatment, medically prescribed supplementary treatments (*e.g.* hydrotherapy, physiotherapy, massages), adjuvants, costs related to periods of disability, medication and intervention related costs.

Specific medical costs refer to the costs of the diagnoses CVD, DMT2, hip and/or knee OA, OW/OB and the intervention costs.

##### Process evaluation

Over the whole study period, the intervention will be evaluated from the perspective of interventionists, participants, study sites and involved organizations in the form of questionnaires, qualitative interviews (QI), documentation and notation sheets and telephone screenings.

To compare the intervention program with the characteristics of the standard care arm, the control group will be asked questions on the utilization of standard care offers.

Participants’ satisfaction with *MultiPill-Exercise* will be evaluated using a modified version of the ZUF-8 within the intervention group [54, 87]. In order to adapt the 8-item instrument (4-point Likert scale) to the outpatient setting, minor modifications were made. In addition, qualitative interviews will be conducted with participants of the intervention group after program completion, including questions on the appropriateness and quality of the intervention delivery, with the aim of gaining greater insight into participants' views on the intervention.

To assess treatment fidelity, standardized notation sheets will be used for the various intervention modules (exercise, participant education session, individual counselling sessions). Interventionists will be instructed to record any deviations concerning the intended intervention delivery. Further, the study team will document any questions of the interventionists regarding the program delivery. This kind of ‘troubleshooting’ during the intervention phases will not only ensure high treatment fidelity but also provides information on possible barriers and facilitators of the intervention delivery.

To capture characteristics of the interventionists, the following questionnaires will be used in versions adapted to *MultiPill-Exercise* before (t0) and after the intervention (t6): First, a German version of the Occupational Self-Efficacy Scale (OCCSEFF) [88] modified according to Rigotti, Schyns [55]; second, a scale transferred from the school context assessing subjective task values [56]; and third, we use items of the three implementation outcome measures according to Weiner, Lewis [89]: Acceptability of Intervention Measure (AIM), Intervention Appropriateness Measure (IAM), and Feasibility of Intervention Measure (FIM).

In-depth analyses on interventionists’ view on the program will be done based on qualitative interviews with at least seven of the interventionists after the last intervention delivery regarding acceptability, practicality, appropriateness and fidelity aspects of the latter. In addition, their satisfaction with the interventionists’ training regarding the overall structure, contents and methods will be assessed in accordance to a formative evaluation procedure regarding exercise therapist training developed by Göhner, Schagg [58].

The evaluation of implementation regarding interfaces of the health service innovation will be conducted by employees of the health insurance company (APS) with short questions based on the AIM, IAM and FIM [89] regarding the practitioners’ view on the acceptability, appropriateness and feasibility of the program and its prescription. In addition, participants in the control and intervention groups will be asked about their mode of access into the study program during telephone screenings that will take place during recruitment. The evaluation of implementation regarding the interface with community-based exercise offers will be carried out after the last intervention delivery using qualitative interviews with a sub-sample of the participants of the intervention group (14 in total) and at least seven employees of the health insurance company.

##### Adherence

The number of attended training sessions will be documented by the exercise instructors for all on-site trainings. Furthermore, the returned training logs (number and type of exercises, subjective exhaustion) will be evaluated for training adherence.

##### Safety: Adverse events and side-effects

The rating of the safety of the intervention is documented by listing exercise related adverse events or side-effects by different parties: The exercise specialists of the study sites will document all potential adverse events of the exercise of which they have become beware of. Participants are urged to report adverse events or side-effects to their guide or to report them in their training-log. Exercise-related adverse events are also retrospectively assessed via the online questionnaire at t3 and t6. All participants can further contact the study physician of the university hospital at any time as outlined in the participant information sheet. The standard adverse events and severe adverse event form of clinical trials will be implemented in the SecuTrial database.

##### Covariates

The following additional diagnoses, which are not considered as inclusion criteria but are of interest, are also evaluated:

To assess psychological stress of the participants in the context of a physical illness, the German Version of the 14-item Hospital Anxiety and Depression Scale (HADS-D) will be used [59]. The questionnaire shows good validity and reliability [90].

The German version of the osteoporosis risk test (ORT) will be applied to screen for the current state of osteoporosis risk [60].

To assess the severity of back pain retrospectively over three months, a reliable and valid German version of the chronic pain grade (CPG) will be used [61, 62].

### Statistical analysis

The primary endpoint of the statistical analysis is the HEPA Index [73]. It will be analyzed at t6 immediately after the termination of Phase 2 of the intervention using a baseline adjusted analysis of covariance with primary factor “intervention” and including study site and recruiting phase as covariates. In case of a non-significant result, the confirmatory part of the analysis stops and all remaining analyses of the primary endpoint are non-confirmatory. In case of a significant result, the same type of model will be run for t12 as well as t18 and interpreted as confirmatory. This hierarchical approach avoids corrections for multiple testing. This seems justified, as we may expect that the effect of the intervention decreases during the follow up time.

Continuous secondary endpoints will be analyzed using the same statistical methods (analysis of ANCOVA, if baseline values are available, analysis of variance otherwise), but results will not be interpreted as confirmatory even if *p*-values and 95% confidence intervals of the main effects of the intervention are given. Binary outcomes will be analyzed using similar logistic regression models. This also holds for adherence to the intervention.

In each analysis, interactions between study site and intervention will be examined and included in the model if necessary. Success of randomization will be assessed using baseline comparisons between both study arms. Exploratory analysis will inspect the prognostic validity of the PROCAM-Score and specific diagnoses. Specific disease related outcomes will be analyzed in the respective subgroups only (section “ secondary and other outcome measures”, paragraph (3)).

Population treatment will be the intention of the primary analysis population. This population includes all participants who contribute at least baseline values of the primary outcome. Multiple imputations will be applied to subjects who drop out or do not contribute measurements of the primary outcome for other reasons.

An additional analysis is planned for the sub-sample as described in paragraph (5) of this section: laboratory values (blood, urine) will be analyzed using log2 transformation for qPCR data (fold changes). Data will be checked for normal distribution and subsequently analyzed for differences between the two groups using one-way ANOVA or Kruskal–Wallis test, respectively.

#### Sample size

The primary endpoint is the HEPA index from the EHIS-PAQ. For this index we have raw data from the “Gesundheit in Deutschland” GEDA 2014/2015 study [91]. The standard deviation for weekly minutes of activity is 289 for male subjects and 233 for female subjects. A relevant improvement of activity is set to 15 min of activity per day. This corresponds to 105 min per week and an effect size of 0.36 conservatively using the standard deviation for male subjects. The sample size estimation is done for a t-test for independent samples. We need 123 evaluable subjects per group to achieve a power of 80% with a type 1 error of 0.05 (two-sided). With 320 allocated subjects and assuming 20% drop outs in both groups, we expect 256 subjects with primary endpoint. Taking into account a loss of seven degrees of freedom due to adjustment of study site and two degrees of freedom due to adjustment of baseline and gender, we achieve the number of 123 evaluable participants per group. However, we assume that due to the baseline adjustment the actual power in the ANCOVA model will be larger than calculated.

#### Economic evaluation

The costs (including the costs for the intervention) will be related to the differences between the groups in quality adjusted life years (QALYs) (Eq. 1) and health related effects (e.g. HOOS Index, PROCAM-Score: Eq. 2).

Equation 1: Cost-Utility Analysis (CUA) = Incremental Cost Utility Ratio (ICUR)

1 $$\mathrm{ICUR }=\frac{\Delta Cost}{\Delta QALYs}$$

Equation 2: Cost-Effectiveness Analysis (CEA) = Incremental Cost-Effectiveness Ratio (ICER)

2 $$\mathrm{ICER }=\frac{\Delta Cost}{\Delta Effect}$$

Uncertainty sampling in the ICER will be handled using nonparametric bootstrapping and graphically presented on a cost-effectiveness plane. We will examine cost-effectiveness and cost-utility from a societal and health care perspective.

#### Data monitoring

We plan for internal monitoring from a physician not involved in data assessment, data management and data analysis, independent from the sponsor and without competing interests. The monitoring will be conducted referring to the standard operating procedure for data monitoring provided by the Centre for Clinical Trials of the Medical Faculty and the University Hospital. The physician will be certified as a Good Clinical Practice (GCP)-physician. The monitor will undertake patient validation and source data verification including a complete check of the documentation of inclusion and exclusion criteria and informed consent and the appropriateness of study inclusion according to the presence and absence of inclusion and exclusion criteria. Further source data that have be transferred into the eCRF will be checked at random (approx. 10%).

## Discussion

Within this protocol, a comprehensive PA promotion program for people with multiple NCDs is introduced. *MultiPill-Exercise* has been designed to address insufficient active people suffering of multiple NCDs, including a holistic approach. Several research questions will be evaluated: (1) The effectiveness of a comprehensive lifestyle and exercise intervention specifically designed for patients with multimorbidity on physical activity status (primary outcome of the trial); (2) the effectiveness of the intervention on generic and disease specific health outcomes, (3) the effectiveness of the intervention regarding the prevention and progression of specific diseases (4); the economic efficiency of the lifestyle intervention in the treatment of several NCDs; (5) the feasibility of implementation of the intervention into patient care in a regular health care setting including safety aspects of the intervention. (1) to (4) will be evaluated in the short and long term in comparison to typical health care offers.

### Limitations

Several limitations have to be addressed. All participants are allowed to take part in other health care offers. This includes IG and CG. Also, the number of health offers for CG is not completely limited. This cannot be prohibited, as all insurance holders with a physician’s recommendation have the right to participate in several health care offers. The exclusive participation in *MultiPill-Exercise* only cannot be ensured and, as stated, the number of offers for the CG cannot be completely predicted. To account for this, all follow-up questionnaires ask after any additional participation in any health care offer.

The weighting of nutrition and exercise in the intervention is not equal in this study, as the primary focus of the intervention is on exercise. Nevertheless, both components are an elementary part of comprehensive intervention.

Although follow-up data will be gathered in the context of this trial for 18-months, statements on the effect of the intervention in the long run, such as rates of post-study surgery and disease specific health care costs, may be limited. If the 18-month evaluations are positive in terms of study outcomes, a protocol amendment for a 5-year follow up may be considered.

## Conclusion

Results of this pragmatic trial will assess the effectiveness, efficiency and safety of a comprehensive physical exercise intervention program in people with multiple chronic diseases in a real-life scenario. This pragmatic trial will therefore be used as a proof of concept with the opportunity of a direct translation of the program into clinical routine in the case of its positive evaluation. Both, the translational approach on the one hand and the multi-site RCT with its rigor methods and standardized operating procedures for the conduction of the intervention on the other hand, will allow valid conclusions for the implementation of physical exercise interventions in people with multimorbidity.

## Supplementary Information

**Additional file 1.**

**Additional file 2.**

**Additional file 3.**

**Additional file 4.**

## Data Availability

Data and material used and/or analyzed during the current study will be made available from the corresponding author on reasonable request after the conclusion of the study. The study results will be published both as publications and within the framework of national and international congresses. The study was also registered in the German Clinical Trial register, which is open to the public. If the study is successful, the *MultiPill-Exercise* program will be established in the health care system of the statutory health insurance company in the long term.

## Abbreviations

AIM: Acceptability of Intervention Measure
APS: Physician-Partner-Service (Arzt-Partner-Service)
BMI: Body Mass Index
BMZI: Bernese Motive and Goal Inventory
BW: Baden-Wuerttemberg
CEA: Cost-Effectiveness Analysis
CG: Control Group
COMET: Counseling approach based on motives and goals in exercise and sport
CPG: Chronic pain grad
CUA: Cost-Utility Analysis
CVD: Cardiovascular disease
DEGS: German Health Examination Survey
DMP: Disease management program
DMT2: Diabetes mellitus type 2
EHIS-PAQ: European Health Interview Survey – Physical Activity Questionnaire
eCRF: Electronic case report form
FIM: Feasibility of Intervention Measure
FFQ: Food frequency questionnaire
GCP: Good Clinical Practice
GDRS: German Diabetes Risk Score
GEDA study: German Health study
HADS-D: Hospital Anxiety and Depression Scale- German
HEPA Index: Health enhancing physical activity index
HOOS: Hip Osteoarthritis Outcome Score
IAM: Intervention Appropriateness Measure
ICER: Incremental Cost-Effectiveness Ratio
ICF: International Classification of Functioning, Disability and Health
ICUR: Incremental Cost Utility Ratio
IG: Intervention Group
KOOS: Knee injury and Osteoarthritis Outcome Score
MSES: Multidimensional Self-Efficacy for Exercise Scale
NCD: Non-communicable disease
OCCSEFF: Occupational Self-Efficacy Scale
OA: Osteoarthrosis
OB: Obesity
ORT: Osteoporosis Risk Test
OW: Overweight
PA: Physical Activity
PAHCO: Physical activity-related health competence
PE: Physical Exercise
PRECIS: Pragmatic-Explanatory Continuum Indicator Summary
PROCAM-Score: Prospective Cardiovascular Muenster Study-Score
QALY: Quality adjusted life years
RCT: Randomized controlled trial
SKK-Scale: Self-concordance of sport- and exercise-related goals scale
SPIRIT: Standard Protocol Items: Recommendations for interventional trials
TSK-GV: Tampa Scale for Kinesoiophobia – German version
VR12: Veterans RAND 12 Item Health Survey
