# Supplementary figures and images for: Implementation and evaluation of an individualized physical exercise promotion program in people with manifested risk factors for multimorbidity (MultiPill-Exercise): a study protocol for a pragmatic randomized controlled trial
Source: BMC Public Health. 2022 Jun 13;22:1174. doi: 10.1186/s12889-022-13400-9 (PMC9190168; doi:10.1186/s12889-022-13400-9)

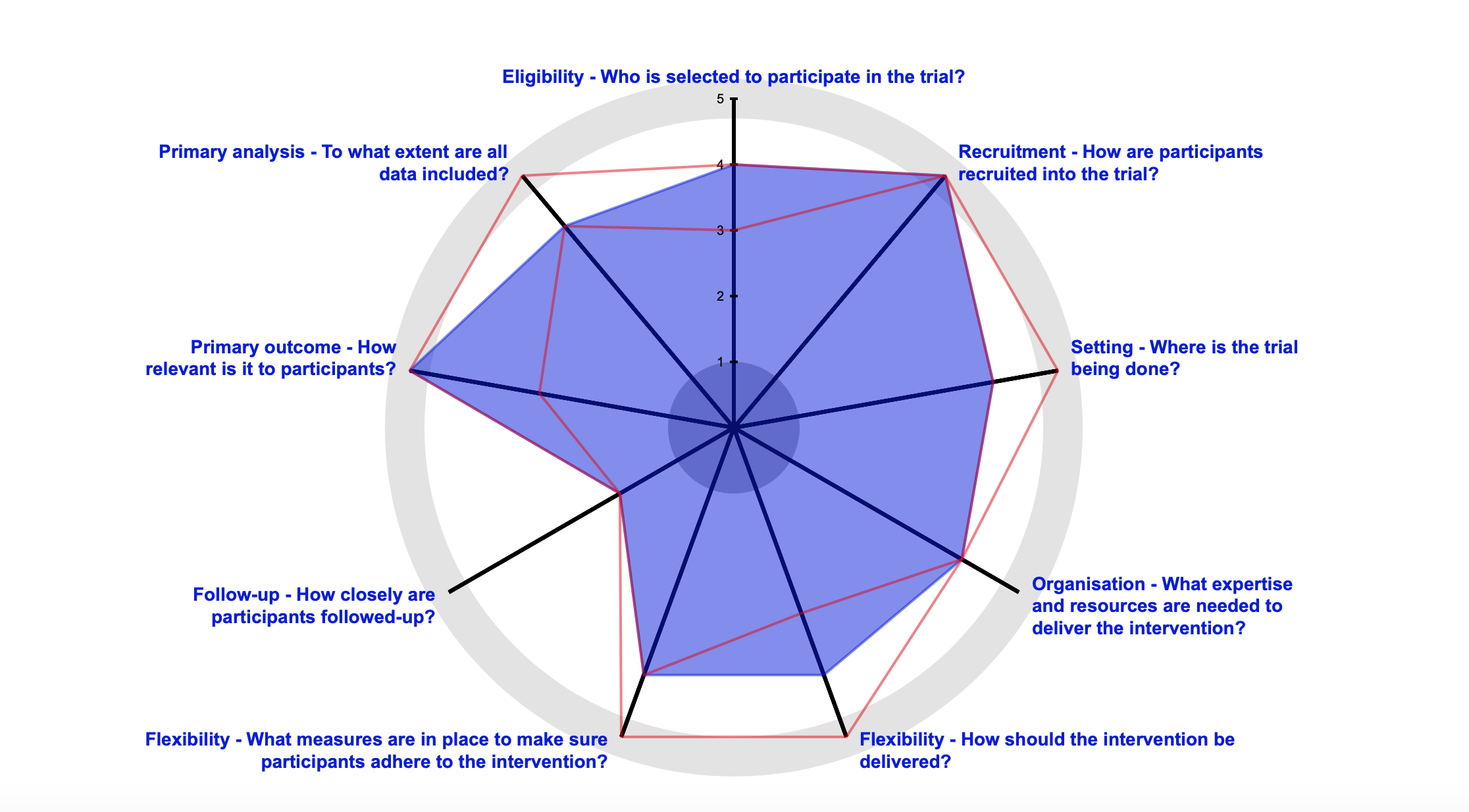

Supplement: Supplementary file 2 — Additional file 2. [file 12889_2022_13400_MOESM2_ESM.png]
