# Supplementary material for: Implementation and evaluation of an individualized physical exercise promotion program in people with manifested risk factors for multimorbidity (MultiPill-Exercise): a study protocol for a pragmatic randomized controlled trial
Source: BMC Public Health. 2022 Jun 13;22:1174. doi: 10.1186/s12889-022-13400-9 (PMC9190168; doi:10.1186/s12889-022-13400-9)
Supplement: Supplementary file 3 — Additional file 3. [file 12889_2022_13400_MOESM3_ESM.docx]

Table 1: Intervention components MultiPill-Exercise

| Designation | Content/Technique | Rational | Setting (how) | Dosage  Phase 1 | Dosage  Phase 2 | Reference |
| --- | --- | --- | --- | --- | --- | --- |
| Endurance training | - Basic endurance training - Endurance oriented interval training | Improving endurance performance using different training methods  W1-2: 60% max HR  W3-12:   1. moderate: 70% max. HR 2. EIT: 4 minutes 90% max. HR – 4 minutes 30 Watts | SS or HB, indoor or outdoor, group or individual | At least 2x/ week, total duration: 90-150 minutes | At least 2-3x/week, total duration 150 minutes | (1-4) |
| Machine-based strength training | Strength training using weight-machines | Movement learning W 1-2: 30% maximum strength (S 1 - R 30)  Strength endurance W 3-6: 30-40% of maximum strength (S 2- R 25)  Muscle building training W 7-12: 70% of maximum strength (S 3 - R 15) | SS, individual | At least 1x/week, duration: approx. 90 minutes |  | (1, 3, 4) |
| Functional training | Whole body strengthening exercises with own body weight | Strength perseverance training  W 1-12: S 2 - R 25 | HB, Video instruction | At least 1x/week, duration 30 minutes | 1-2x/week | (1, 3, 4) |
| Movement Teaser | Activity offers including the following motives:   - Experience nature - Rhythm and Aesthetics - Relaxation - Distraction - Fitness and Health | Getting to know various types of exercise to identify personal exercise motives and goals | SS, Indoor or Outdoor, group training | 4 units, each 60 minutes |  | COMET (5) |
| Participant education session, delivering theoretical and practical knowledge | Competence approach (learning, reflecting, experiencing)   - Training principles - Dietetics - Motivation, Volition and Barriers - Motives and goals of sport - Active everyday life | Promotion of physical activity-related health competence (especially knowledge of effects of PE and Action planning) | SS, group training | 5 units each 30 minutes | 1 unit, 30-45 minutes | MoVo (6)  BMZI (7)  Active everyday life (8, 9) |
| Training log | BCT (motivational)   - Weekly targets and feedback | Action and coping planning for long-term lifestyle change. | HB and SS | 1/week | 1/week | (10, 11) |
| Individual counselling sessions | MI, BCT   - Training log - Facilitators and barriers to sport continuation - Motives and goals in sport - Nutrition and Dietetics | Enhancing motivation and volition to regular PE in the long term. Identifying facilitators and barriers related to sport participation.  Knowledge on healthy and anti-inflammatory nutrition. | SS | 3 | 2 | MI (12)  MoVo (6)  COMET (5)  Anti-inflammatory diet (13) |
| Disease-specific offers | - Relaxation (CVD) - Special exercises for M. quadriceps femoris/Mm. glutei (OA) | Additional disease-specific offers to enhance health benefits. | SS and HB | Optional: 1-2 offers/person |  | (14, 15) |
| BCT = Behavior change techniques, BMZI = Bernese motive and goal inventory, COMET = Counseling approach based on motives and goals in exercise and sport, CVD = Cardiovascular diseases, DMT2 = Diabetes Mellitus Type 2, HB = Home based, HR= Heart rate, EIT = Endurance oriented interval training, MI = Motivational interviewing, OA = Osteoarthritis, OB = Obesity, OW = Overweight, R = Repetitions, S = Sets, SS = study site, W = Week | | | | | | |

1. Bull FC, Al-Ansari SS, Biddle S, Borodulin K, Buman MP, Cardon G, et al. World Health Organization 2020 guidelines on physical activity and sedentary behaviour. British journal of sports medicine. 2020;54(24):1451-62.

2. Taylor JL, Holland DJ, Spathis JG, Beetham KS, Wisløff U, Keating SE, et al. Guidelines for the delivery and monitoring of high intensity interval training in clinical populations. Prog Cardiovasc Dis. 2019;62(2):140-6.

3. Rütten A, Pfeifer K. National Recommendations for Physical Activity and Physical Activity Promotion. Rütten A, Pfeifer K, Abu-Omar K, Geidl W, Messing S, Burlacu I, et al., editors. Erlangen: FAU University Press; 2016.

4. Nieß AM, Thiel A. Körperliche Aktivität und Sport bei Typ-2-Diabetes. Diabetologie. 2017;12:112-26.

5. Schmid J, Schorno N, Gut V, Sudeck G, Conzelmann A. “What type of activity suits me?” Development and implementation of the Exercise and Sport Counselling Approach COMET. Zeitschr Sportpsychol. 2020;27:127-38.

6. Fuchs R, Goehner W, Seelig H. Long-term effects of a psychological group intervention on physical exercise and health: the MoVo concept. Journal of physical activity & health. 2011;8(6):794-803.

7. Schmid J, Gut V, Conzelmann A, Sudeck G. Bernese motive and goal inventory in exercise and sport: Validation of an updated version of the questionnaire. PLoS One. 2018;13(2):e0193214.

8. Sudeck G, Höner O, Edel K. Integration theoriegeleiteter edukativer Maßnahmen in die kardiologische Sport und Bewegungstherapie. Bewegungstherapie und Gesundheitssport. 2007;22:94-8.

9. Pfeifer K, Sudeck G, Geidl W, Tallner A. Bewegungsförderung und Sport in der Neurologie–Kompetenzorientierung und Nachhaltigkeit. Neurol Rehabil. 2013;19(1):7-19.

10. Fuchs R. Aufbau eines körperlich-aktiven Lebensstils : Theorie, Empirie und Praxis. 1. Aufl. ed. Fuchs R, Göhner W, Seelig H, editors. Hildesheim: Hogrefe; 2007.

11. Geidl W, Hofmann J, Gohner W, Sudeck G, Pfeifer K. Behaviour-orientated exercise therapy-initiating and maintaining a physically active lifestyle. Rehabilitation (Stuttg). 2012;51(4):259-68.

12. Miller WR, Rollnick S. Motivierende Gesprächsführung: Motivational Interviewing: 3. Auflage des Standardwerks in Deutsch: Lambertus-Verlag; 2015.

13. Esposito K, Pontillo A, Di Palo C, Giugliano G, Masella M, Marfella R, et al. Effect of weight loss and lifestyle changes on vascular inflammatory markers in obese women: a randomized trial. Jama. 2003;289(14):1799-804.

14. Bennell KL, Dobson F, Hinman RS. Exercise in osteoarthritis: Moving from prescription to adherence. Best Practice & Research Clinical Rheumatology. 2014;28(1):93-117.

15. Liu D, Yi L, Sheng M, Wang G, Zou Y. The Efficacy of Tai Chi and Qigong Exercises on Blood Pressure and Blood Levels of Nitric Oxide and Endothelin-1 in Patients with Essential Hypertension: A Systematic Review and Meta-Analysis of Randomized Controlled Trials. Evidence-Based Complementary and Alternative Medicine. 2020;2020:3267971.
